# Dendritic synaptome of calcium-binding protein containing GABAergic interneurons in the mouse primary visual cortex

**DOI:** 10.3389/fncir.2025.1644572

**Published:** 2025-10-08

**Authors:** Petra Talapka, Zsolt Kocsis, Lívia Diána Marsi, Vera Etelka Szarvas, Zoltán Kisvárday

**Affiliations:** ^1^HUN-REN Neuroscience Research Group, University of Debrecen, Debrecen, Hungary; ^2^Department of Anatomy, Histology and Embryology, Faculty of Medicine, University of Debrecen, Debrecen, Hungary

**Keywords:** electron microscopy, neocortex, dendritic input synapses, GABAergic neurons, 3D reconstruction, calcium binding proteins (CBPs)

## Abstract

This article aims to provide a synaptic input database called, dendritic synaptome for dendrites of calcium-binding protein-containing interneurons [calbindin-D28K (CB+), calretinin (CR+), parvalbumin (PV+)] employing a modified correlated light and EM method, the “mirror-technique” that allows for investigating neuronal compartments while preserving utmost ultrastructural quality (Talapka et al., 2021). Nine dendrites and all presynaptic boutons (*n* = 815) impinging on their surface were traced and reconstructed in three-dimensions (3D) using serial section transmission electron microscopy (ssTEM). The following basic parameters of the synapses were determined: The ratio of symmetric (“ss” or putative inhibitory) and asymmetric (“as” or putative excitatory) synapses, the number of synapses per unit length of dendrite (i.e., density of “as” and “ss”), surface area and volume of presynaptic boutons, and area of the active zones of synapses. Significant differences in the morphometric parameters of asymmetric, but not in symmetric, synapses were detected between the three interneuron subtypes. Surface extent and the number of synapses on PV+ dendrites were the largest compared to the other two subtypes. Although the distribution of presynaptic boutons differed between dendrites, clustering of the presynaptic boutons could be revealed only for PV+ dendrites. Based on our serial-section electron microscopy (ssEM) reconstructions and corresponding light microscopy (LM) databases of CBP dendrites, it was calculated that on average a single CB+, CR+, and PV+ interneuron receives 2,136, 2,148, and 2,589 synapses, respectively, of which 74.6, 81.5, and 85.3% are excitatory, that is, asymmetric, and the remaining inhibitory, that is, symmetric. Carriage return findings provide essential quantitative information to establish realistic computational models for studying the synaptic function of neuronal ensembles in the mouse primary visual cortex.

## Introduction

In the simplest form, cortical circuits are comprised of glutamatergic excitatory projection neurons (PCs) and local gamma-aminobutyric acid (GABA)ergic inhibitory interneurons (INs), which together regulate signal flow and shape overall network dynamics. Regulation of the intracellular Ca^2+^ concentration is crucial for synaptic transmission and its short-term and long-term modulation, which cannot occur without the buffering capacity of cytosolic calcium-binding proteins (CBPs) (Catterall and Few, 2008; Clapham, 2007; Schwaller, 2009; Xu-Friedman and Regehr, 2004). In the primary visual cortex (VISp), the CBP-containing INs represent a morphologically diverse population, approximately 95% of all inhibitory neuron types (DeFelipe et al., 1999; Disney and Aoki, 2008).

Most fast-spiking basket cells belong to parvalbumin immunopositive (PV+) INs, the largest IN population, which contribute to network synchrony and regulate the coactivity of neuronal ensembles (Rudy et al., 2011; Agetsuma et al., 2017). PV + INs represent one of the three non-overlapping populations that form local microcircuits with the PCs [besides vasoactive intestinal polypeptide (VIP) and somatostatin (SOM)], and their functional and morphological properties are widely investigated (Tasic et al., 2016; Tremblay et al., 2016; Hertäg and Sprekeler, 2019). Calretinin immunopositive (CR+) INs can be categorized into two major forms based on their dendrite arborization patterns: bipolar and multipolar (Camillo et al., 2018; Xu et al., 2010). They co-localized with VIP or SOM and elicit either a disinhibitory effect (Meskenaite, 1997; Gonchar and Burkhalter, 1999) or an effect that leads to a net inhibition of cortical activity (Camillo et al., 2018). For the third IN class, calbindin-D_28K_ (CB)-containing neurons, limited knowledge and quantitative datasets regarding their density and distribution in the rodent brain are available to date (DeFelipe, 1997; Frantz and Tobin, 1994; Bjerke et al., 2020). CB is mainly expressed in INs with double-bouquet characteristics, but the protein has also been detected in PCs, for instance, in the medial entorhinal cortex and in the hippocampal CA1 region (Ray et al., 2014; Merino-Serrais et al., 2020). Nevertheless, the functional role of this subtype is still elusive (Li et al., 2017; Harris et al., 2016).

Previous theoretical studies showed that recurrent activity among cortical neurons that are similarly tuned to a stimulus property, such as visual orientation, could increase the response gain of the cells, thereby amplifying the response to thalamic input as well as sharpening the response selectivity or increasing signal-to-noise ratio (Ben-Yishai et al., 1995; Douglas et al., 1995; Suarez et al., 1995; Chance et al., 1999). A direct correlation has been demonstrated recently in layer 2/3 (L2/3) PCs between dendritic morphology and direction, as well as orientation selectivity in the mouse VISp (Weiler et al., 2022).

Despite the vast amount of literature on the various properties of IN types, there is still not available a suitable realistic neuronal model, which enables us to study these connections because accurate morphometric data regarding the composition and morphometric parameters of synaptic inputs of CBP-containing INs are almost completely missing (Gulyás et al., 1999; Hwang et al., 2021). The location of synaptic inputs in relation to the soma (cell body) is critical in this context, along with the excitatory and inhibitory balance (E/I balance) inputs that impinge on the dendrites. According to a comprehensive ultrastructural study mapping mainly the perisomatic region of different neuron types (i.e., PCs, SST+, and PV+) in the mouse VISp, overall excitatory or inhibitory outputs of the VISp are supposed to be balanced by the cell-type-specific composition of synapses (Hwang et al., 2021).

Here we examined the synaptic parameters of dendrites belonging to the CBP-containing INs accomplished with the help of the mirror technique (Talapka et al., 2021). Three dendrites of each subtype were analyzed from soma origin using serial section EM. The quantitative features of the dendrites and the synapses established on their surface offer a substantial promise for modeling approaches in deciphering the functional role of their interaction.

## Materials and methods

### Experimental animals

Adult 10-week-old male C57Bl/6J mice were used for the experiments. Animals were maintained and bred in the animal house facility of the Department of Anatomy, Histology and Embryology under controlled conditions according to the guidelines for the care and use of laboratory animals approved by the Animal Research Committee of the University of Debrecen (License Nr. 12/2016/DEMAB) in accordance with the Hungarian Enactment for the Use of Laboratory Animals (40/2013.(II.14.) Gov.HUN) and the European Union guidelines for the care of laboratory animals (Directive 2010/63/EU).

### Identification of calcium-binding containing GABAergic interneurons with the “mirror-technique”

Tissue samples were derived from 15 animals, five mice for each marker. For pre-embedding immunostaining (IS) and designation of interneurons (INs) in the primary visual cortex (VISp), tissue fixation was performed using the protocol described earlier (Talapka et al., 2021). Briefly, mice were transcardially perfused using a fixative containing 2% paraformaldehyde, 1% glutaraldehyde, and 15% (v/v) saturated picric acid in 0.1 M phosphate buffer (PB; pH = 7.4) for 40 min after 1 min washing with Tyrode’s solution. Brains were removed and stored in the same fixative at 4°C overnight. Then, serial vibratome sections (60 μm, VT-1000S, Leica) were made in the frontal plane from tissue blocks containing the VISp (see Allen Mouse Brain Atlas;^1^ Lein et al., 2007).

**Figure 1:**
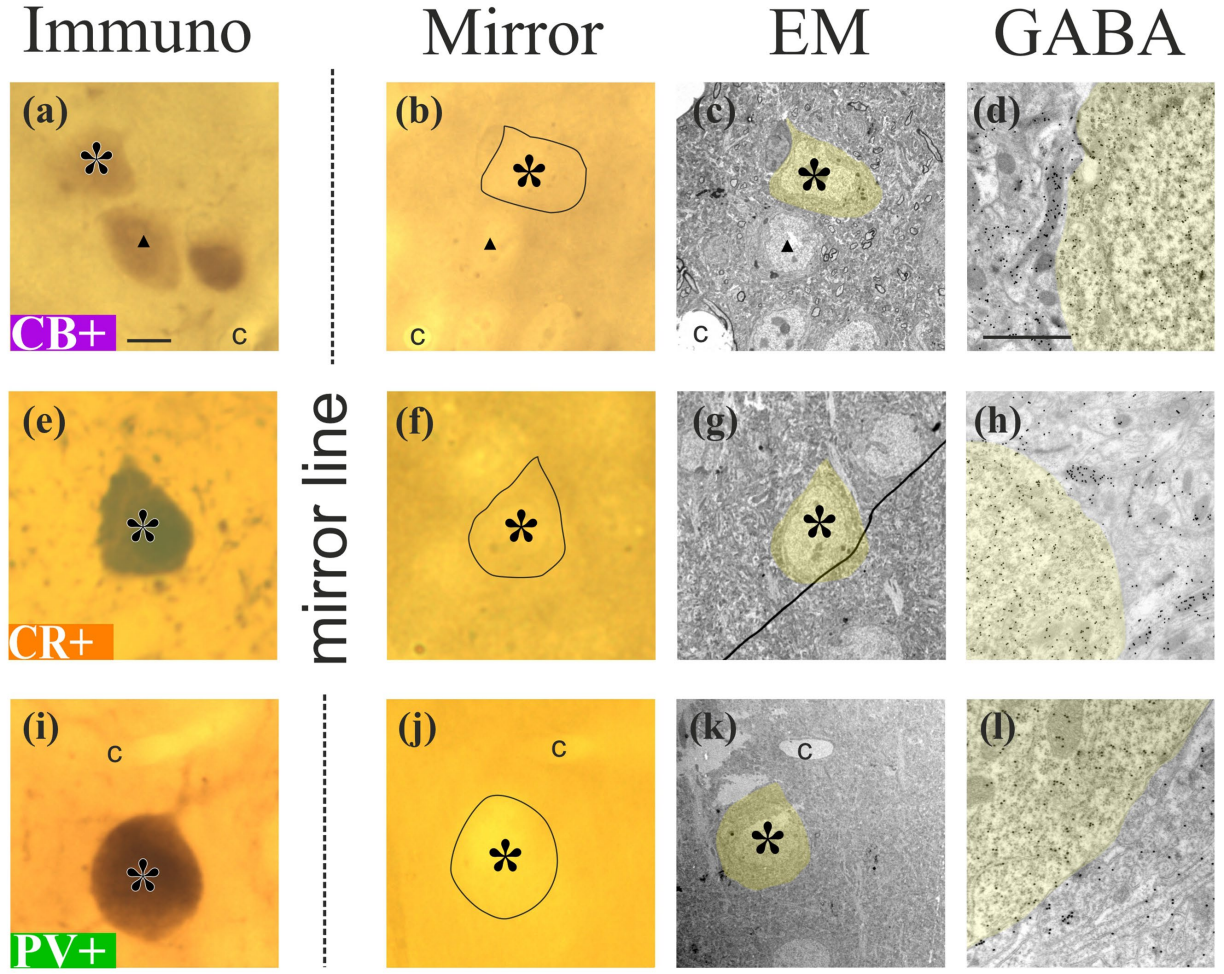
LM-EM correlation of the three calcium-binding protein-containing interneuron (IN) types by the “mirror-technique.” Exemplary images of a calbindin-D_28K_ immunopositive [CB, **(a–d)**], a calretinin immunopositive [CR, **(e–h)**], and a parvalbumin immunopositive [PV, **(i–l)**] neuron (asterisk) were chosen to be studied. In the left column, light micrographs represent the surface of immunostained sections [immuno, **(a,e,i)**], the second column represents the mirror surface of non-immunostained sections [mirror, **(b,f,j)**], both containing the same somata (black contours) along the sectioning plane. For correlating the LM images, a few landmarks that fall in the sectioning plane, such as other neuronal somata (triangle) and blood capillaries **(c)**, are also marked. In the third column [EM, **(c,g,k)**], electron micrographs show the location of the neurons (yellow shade) on the surface of the resin-embedded tissue blocks obtained from the non-immunostained sections. Their inhibitory character was confirmed in the cell body using post-embedding immunogold labeling against GABA. The fourth column **(d,h,l)** shows an enlarged view of the cell bodies displaying a high density of gold particles in the soma (yellow shading). Note that the EM sections derive from the surface of the block from where serial section tracing and reconstruction of dendrites began. Scale bar for panels of columns 1–3: 5 μm; panels of column 4: 1 μm.

For immunostaining (IS), monoclonal calbindin antibodies, as well as polyclonal calretinin and parvalbumin antibodies, were used in separate animals to label respective interneuron populations (Table 1). From each tissue block, alternate sections were subjected to either immunohistochemistry (Figures 1a,e,i) or processed for electron microscopy without immunohistochemistry (Figures 1b,f,j). For IS, the sections were incubated with biotinylated secondary antisera for 4 h at room temperature. Following an overnight incubation in avidin-biotin complexed to horseradish peroxidase (1:400 dilution; ABC, Vector Laboratories, Inc., USA, PK-6100), the labeling was visualized by incubating the sections in 3,3-diaminobenzidine-tetrahydrochloride (DAB, 0.05 M in 0.05 M tris buffer pH = 7.6; Merck, Germany, D5637) for 10 min. The enzymatic reaction was completed in the presence of 0.02% H_2_O_2_ for 1–2 min. Finally, sections were post-fixed in 1% osmium tetroxide (OsO_4_; SPI Supplies, USA, 02601-AB) for 10 min, dehydrated in an ascending series of ethanol, and flat-embedded in resin (DurcupanTM ACM; Merck, Germany, 44,610) on slides (Somogyi and Freund, 1989). Alternate sections underwent processing for transmission electron microscopy (TEM). These sections, which were non-immunostained (nIS), received post-fixation in 1% osmium tetroxide (SPI Supplies, USA, 02601-AB) for 30 min. Dehydration and embedding processes were identical to those used for IS samples.

**Table 1:** Summary of antibodies used in pre-embedding immunohistochemistry.

Antibody	Host animal	Dilution	Distributor, category number
Calbindin D-28 K	Mouse	1:2,000	Swant AG, Switzerland; 300
Calretinin	Rabbit	1:2,000	Swant AG, Switzerland; 7697
Parvalbumin	Guinea pig	1:2,000	Synaptic Systems, Germany; 195 004
Anti-mouse secondary antibody	Goat	1:200	Vector Laboratories, Inc., USA; BA-9200
Anti-rabbit secondary antibody	Goat	1:200	Vector Laboratories, Inc., USA; BA-1000
Anti-guinea pig secondary antibody	Goat	1:200	Vector Laboratories, Inc., USA; BA-7000

Immunolabelled cell bodies were selected on the very surface of IS sections. Care was taken to choose only those somata that were sectioned by the vibratome so their complementary parts fell on the surface of the adjoining section treated with osmium alone. The region of interest (ROI) was defined as the chosen IS cell body and other structures on the surface, such as non-IS cell bodies and truncated blood vessels. The ROI was mapped under the light microscope (Leica DMRB, 100 × oil objective), Leica Camera AG, Germany by drawing the contours of the structures using the Neurolucida (version 8.5) neuron reconstruction system. Then the ROI was overlaid with the mirror surface of the adjoining non-IS sections, which allowed us to identify the very same neuronal cell bodies, including the complementary part of the IS soma. Additionally, a thorough photo-documentation was also conducted.

### Preparation of the ROI for electron microscopy

A 1.5 mm × 1.5 mm area of the osmicated section containing the ROIs in the center was cut out from the non-IS section and re-embedded in the block according to the protocol described by Somogyi and Freund (1989). At least three IS somata were selected and prepared for the three inhibitory neuronal markers (PV, CB, and CR). Serial ultrathin sections (50 nm) were cut from the entire thickness of the vibratome sections (60 μm, approximately 1,200 per block) and collected on Formvar-coated (polyvinyl formal; Polysciences, 00631) single slot nickel grids (6–10 sections per grid). Sections were stained with Reynold’s lead citrate solution for 10 min (Reynolds, 1963) to increase contrast. Post-embedding GABA immunogold labeling was applied on approximately every twentieth ultrathin section for validating the GABAergic nature of the selected neurons (soma: Figures 1c,d,g,h,k,l), and for identifying inhibitory presynaptic boutons (Talapka et al., 2021).

### Image acquisition and 3D reconstruction

**Figure 2:**
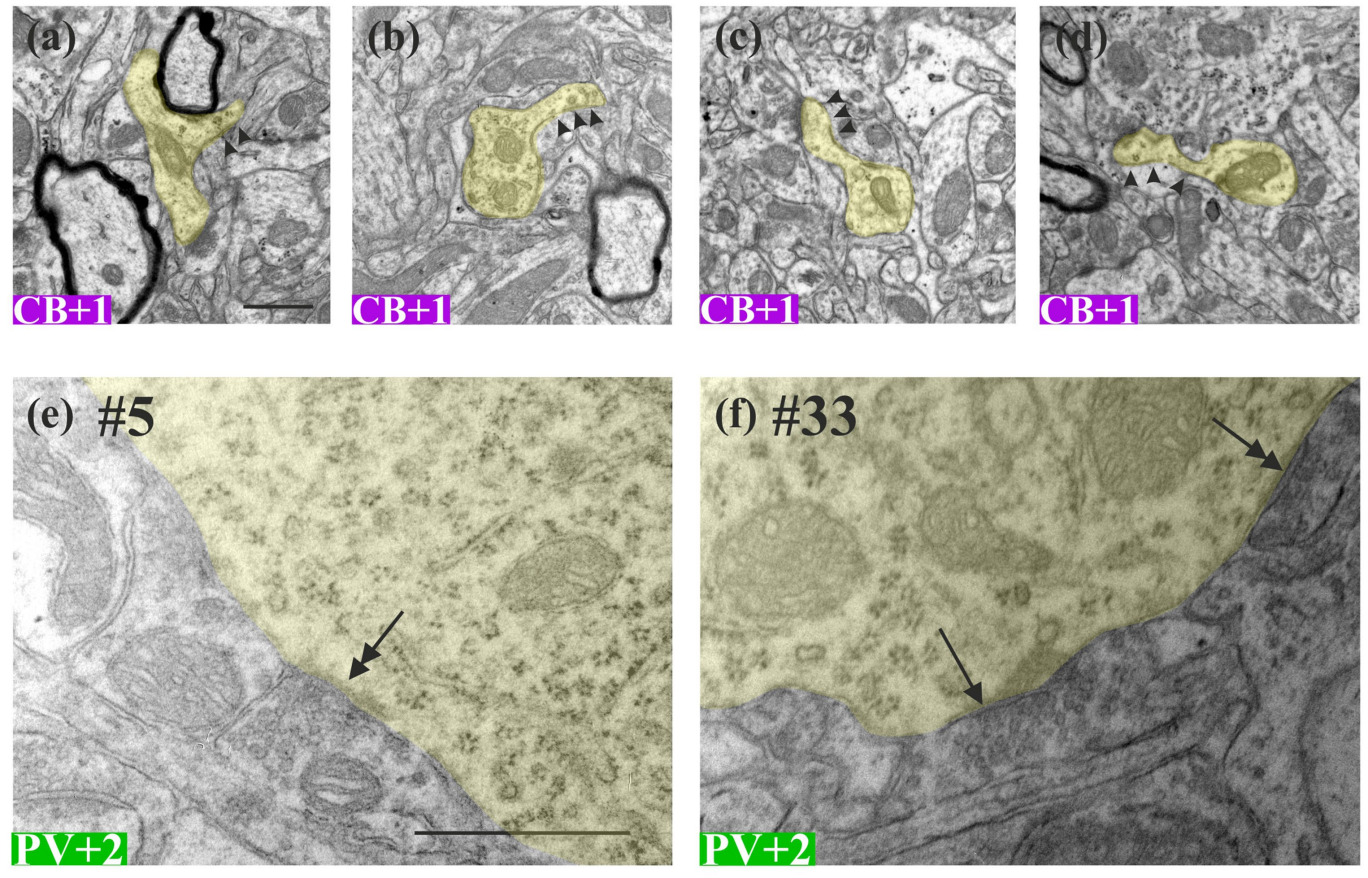
Representative electron microscopic images showing the morphological features of the designated calcium-binding protein containing INs. **(a–d)** Dendrites (yellow) of the calbindin-D_28K_ immunopositive interneuron (CB + 1) had spine-like protrusions (arrow heads) typical of sparsely spiny neurons. **(e,f)** The same parvalbumin-immunopositive soma (PV + 2, yellow shading), presumably a basket cell, receives an asymmetric synapse (double-headed arrow) and a symmetric synapse (arrow) of unknown origin. Numbers (#) indicate ultrathin section number in the serial section stack. Scale bars: **(a–d)** 0.5 μm; **(e)** and **(f)** 1 μm.

Ultrathin sections were examined and photographed using a JEOL-1010 TEM (JEOL Ltd., Japan) equipped with a digital camera (Olympus Veleta). The sections were studied in a sequential order, that is, starting with those representing the mirror surface of the EM block comprising the selected cell body. Subsequent sections toward the opposite surface of the block comprised some dendrites emitted from the same selected cell body (Figures 2a–f). EM images were acquired systematically at two magnifications [low: 10,000 × (7.13 nm/px); high: 30,000 × (2.39 nm/px)] of any given structure, including the dendrites and their presynaptic boutons. Dendrites of selected somata were followed until they terminated or extended beyond the EM block. Attention was made to capture the emergence of the dendrites from the parent cell body.

Using high-power magnification, large objects such as elongated dendrites cannot be viewed in a single image. In this case, an extension of the field of view (FOV) is necessary, so multiple images overlapping with each other must be taken. However, due to non-linear lens distortions, the overlapping image tiles have been corrected using correspondence points, as introduced by Kaynig et al. (2010) in their calibration method.

Three-dimensional volume reconstruction of dendritic shafts and branches, presynaptic boutons, and surfaces of type I or asymmetric (as) and type II or symmetric (ss) synapses was carried out using the TrakEM2 software (graphical interface of Image J 1.46r) as described earlier (Cardona et al., 2012; Talapka et al., 2021). Compression of ultrathin sections perpendicular to the cutting edge was corrected after reconstructions had been completed (Talapka et al., 2021).

### Morphometric analyses

Volume and surface area of dendrites, as well as presynaptic boutons, and surface area of synaptic active zones established by the presynaptic boutons were exported from TrakEM2. Boutons whose reconstruction could not be completed, mainly because of truncated parts of the dendrites, were omitted from statistics.

To determine the volume and surface values of dendrites, first, their diameters were measured on EM images taken at every ~2 μm (~ every 40th 50-nm-thick EM section) along the path. The average diameter was calculated for each dendrite and dendritic branch, respectively. Dendrite path length was obtained using the TrakEM2 and the Reconstruct software packages. The surface area and volume were calculated according to the formula: π × average diameter × path length, and π × (average diameter/2)^2^ × path length. Datasets within each IN type were pooled, and the mean values were depicted.

To determine the accurate location of the synaptic inputs received by dendrites and their distance from the soma, the spatial coordinates (centroid x, y, z) of the synaptic active zones were calculated for each synapse. Since the active zones were present in more than one section, the centroid was obtained in the middle section within the stack. The location of the soma represented the origo (centroid x:0, y:0, z:0) for the distance measurements and was assigned as the first layer of the image stack. The Branched Structure Analyses tool of the Neurolucida software (MicroBrightField, Williston, USA) was utilized to obtain the location and distance values of the synaptic active zones from the soma.

### Centroid calculation for fitting synapse density histogram data

This method identifies the centroid of the synapse density histogram. The centroid is calculated through the following steps. (i) Data filtering excluded any “Not a Number” (NaN) and infinite values, as well as any histogram values that are zero. This ensures that only valid data points are used in the calculation of the centroid. The filtering process involves identifying valid indices where the histogram values are finite and non-zero, and then selecting the corresponding bin centers and histogram values. (ii) The total mass is computed by summing the valid histogram values:


$$\text{total mass}=\sum_{i=1}^{n}h_{i}$$

where h_i_ represents the valid histogram values, and n is the number of these values. If the total mass is zero, it indicates that there are no valid data points to calculate the centroids, and therefore, the centroid coordinates are undefined. (iii) Centroid coordinates calculation: the x-coordinate is calculated as the weighted average of the bin centers, where the weights are the histogram values:


$$x_{c}=\frac{\sum_{i=1}^{n}x_{i}h_{i}}{\text{total mass}\;}$$

and the y-coordinate is determined by the average of the valid histogram values, normalized by the total number of bins in the histogram:


$$y_{c}=\frac{\sum_{i=1}^{n}h_{i}}{\text{total number of bins}}$$

### Statistical analyses

GraphPad Prism (version 8.0.1) and MATLAB (R2022b) were used for the statistical tests. Statistical significance was defined as *p* < 0.05. Shapiro–Wilk’s normality test was performed to determine whether the datasets fit a Gaussian distribution in every case. Statistical comparisons were performed using the Kruskal–Wallis test and with Dunn’s *post hoc* test, too (as the data did not meet the assumptions of normality).

Morphometric parameters of dendrites, presynaptic boutons, and synapses were demonstrated on boxplots. Our aim was to present all boutons and synapses, hence outliers were not excluded. Only boutons and active zones incompletely reconstructed have been omitted from the statistical analyses. The surface/volume ratio was chosen to interpret the quantitative features of presynaptic boutons. In addition to this, excitatory and inhibitory input types were compared to each other within as well as between markers.

For each of the three markers (CB+, CR+, and PV+), cluster analyses of synapses were also made. Notably, K-means clustering supplemented by the elbow method was applied to gain one-dimensional datasets to elucidate the possible spatial organization of synaptic inputs. The distribution was expressed for each marker separately and depicted on scatter plots. Datasets were analyzed within 5-μm bins to avoid noise and ensure gaining the correct results.

## Results

### Morphological features of the CBP interneuron subtypes

#### Calbindin-D_28K_ (CB+) INs

**Figure 3:**
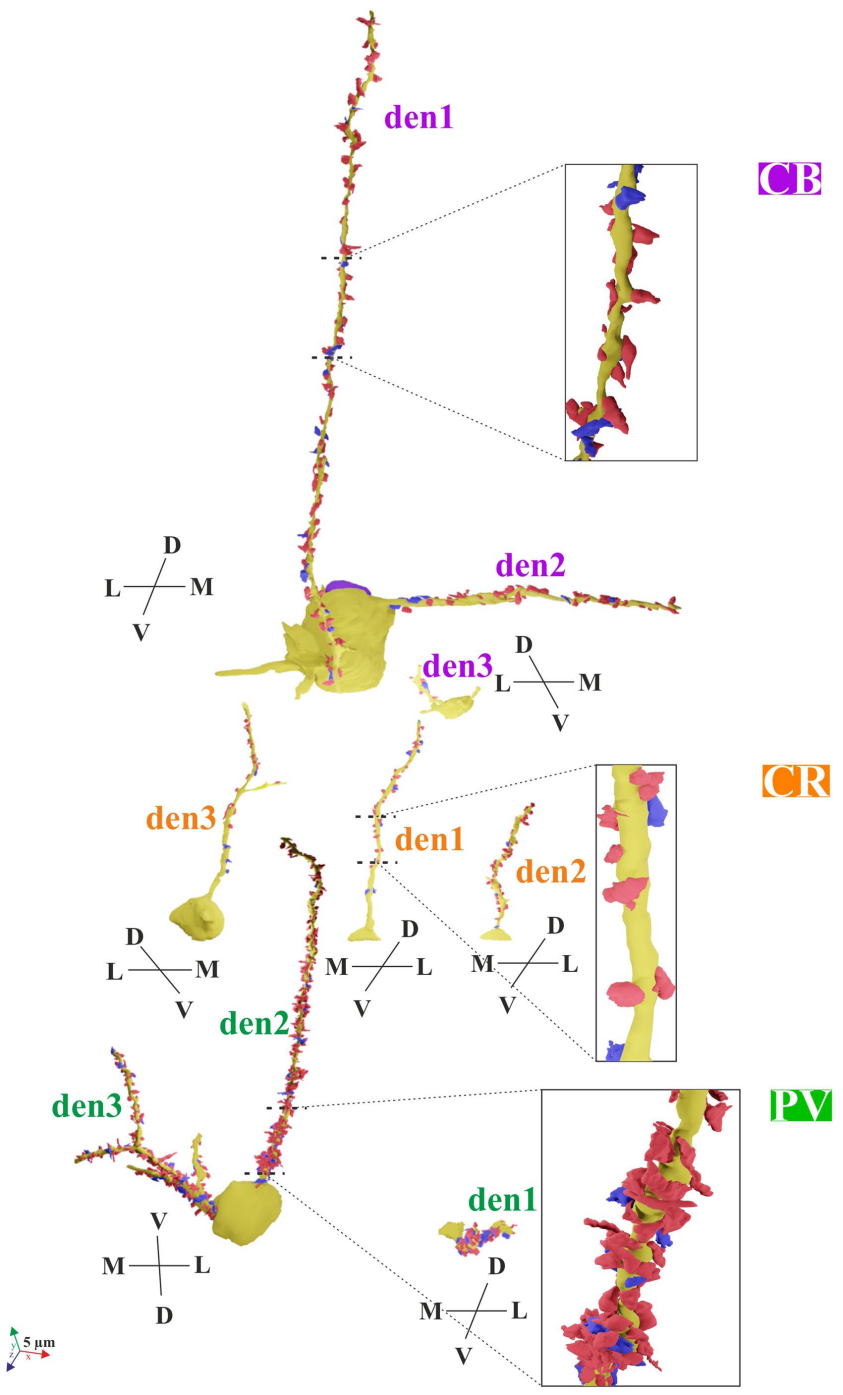
3D reconstructions of the neurochemically labeled interneurons (CB: calbindin-D_28K_; CR: calretinin; PV: parvalbumin). Dendritic segments (den) originated from the somata (yellow) with their presumed inhibitory boutons establishing a symmetric type of synapse (blue) or excitatory boutons establishing an asymmetric type of synapse (red). Zoom-on reconstruction of CB+, CR+, and PV + dendrites is delimited by broken lines. Anatomical directions: dorsoventral (D-V), mediolateral (M-L). Scale bar: 5 μm.

Two neurons were chosen for the CB+ cell population in L5. They were identified as multipolar type, and the dendrites branched several times along their course. From the two labeled cell bodies, altogether six dendrites emerged, of which three were traced at length in the EM. For CB+ 1, two dendrites (Figure 3, CB+ den1,2) were reconstructed from serial EM sections. Den1 ran perpendicular to the sectioning plane, and thus could not be entirely traced because of truncation. Den2 ran parallel with the sectioning plane and could be traced and reconstructed until its terminal end (Table 2 and Figure 3). Notably, CB+ dendrites had a few protrusions reminiscent of dendritic spines without an obvious spine head (Figures 2a–d). All in all, CB + INs could be characterized as a multipolar, sparsely spiny GABAergic subtype. For CB + 2, one dendrite (den3) was traced. Unfortunately, it ran out of the block frame after 200 ultrathin sections; therefore, only its very proximal segment could be reconstructed.

**Table 2:** The number and density of input synapses (as: asymmetric, ss: symmetric) of the dendrites belonging to the designated calcium-binding protein-containing interneurons.

Dendrite ID	Number of EM sections	Number of “as” synapses (%)	Number of “ss” synapses (%)	Number of all synapses (%)	Number of boutons	Length of dendrites (μm)	Synapse density (1/μm)
CB_DEN1	1174	84	24	108	109	125	0.864
CB_DEN2	206	30	6	36	41	58	0.621
CB_DEN3	218	5	1	6	6	15	0.400
Total	**1598**	**119**	**31**	**150**	**156**	**198.0**	**-**
Average	**532**	**39.7** **(79.3)**	**10.3** **(20.7)**	**50** **(100.0)**	**52**	**66**	**0.628**
CR_DEN1	369	34	8	42	43	56	0.750
CR_DEN2	519	46	3	49	50	44	1.114
CR_DEN3	513	22	6	28	28	47	0.596
Total	**1401**	**102**	**17**	**119**	**121**	**147**	**-**
Average	**467**	**34.0** **(85.6)**	**5.7** **(14.4)**	**39.7** **(100.0)**	**40.3**	**49.0**	**0.820**
PV_DEN1	302	48	20	68	72	44	1.545
PV_DEN2	1116	150	28	178	178	155	1.148
PV_DEN3	336	224	26	250	251	221	1.131
Total	**1754**	**422**	**74**	**496**	**501**	**420**	**-**
Average	**584.7**	**140.7** **(85.1)**	**24.7** **(14.9)**	**165.3** **(100.0)**	**167**	**140**	**1.275**
Grand total	**4753**	**643** **(84.0)**	**122** **(16.0)**	**765** **(100.0)**	**778**	**765**	**-**

#### Calretinin immunopositive (CR+) INs

Three neurons were selected in L2/3. They exhibited characteristic morphological features, with dendrites oriented chiefly radially from the soma, that is, toward the pia mater and the white matter. In line with this, in the EM block, only a single radial dendritic shaft originated from the cell body. Furthermore, no dendritic spines or protrusions emerged from the dendrites throughout the entire series of EM sections. The three dendrites could be reconstructed in their entirety since they terminated within the block.

#### Parvalbumin immunopositive (PV+) INs

One PV + IN soma was selected in L5 (PV + 1) and one in L2/3 (PV + 2). Based on light microscopic measurements, these somata were the largest compared with those of the other two types (Figure 1, see left column panels). In the immunostained sections, PV + INs typically emitted several dendrites in all directions, and thus, they belong to the stellate morphological class. By qualitative inspection, the dendrites were usually thicker than those of CB+ and CR + INs. PV+ dendrites were smooth, just like CR + ones bearing no protrusions or spines (not shown).

Commonly, the somata of all CBP types were found to receive both asymmetric (Figure 2e) and symmetric (Figure 2f) types of synapses. In this study, only a qualitative assessment was made regarding the number of somatic synapses. Nevertheless, we confirmed that PV+ cell bodies outnumbered those of the other two cell body types in the synapse density (Gulyás et al., 1999).

### EM features of the dendrites

Summary of the EM findings after shrinkage correction is shown in Table 2. Data of each dendritic shaft is presented together with their side branches. The most prominent differences were observed in the surface/length ratio of dendrites of the different IN types. This ratio represents a good measure of how thick (diameter) an elongated structure is on average (the following mean values were calculated from Table 2: CB+: 1.165; CR+: 1.757; and PV+: 2.144).

**Figure 4:**
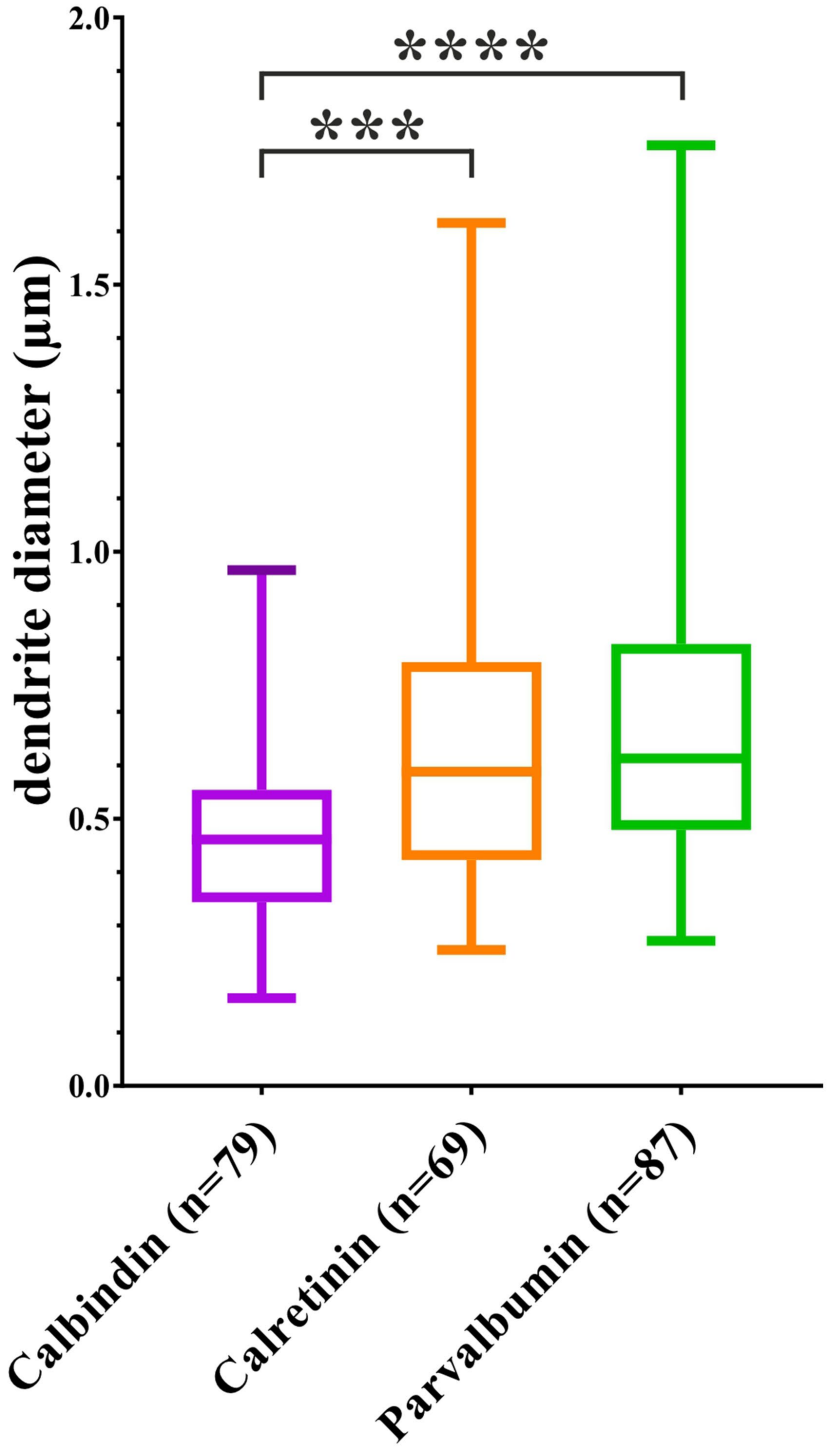
The average diameter of dendrite branches belonging to calcium-binding protein-containing interneurons. The mean diameter of CB + dendrites was the smallest and differed significantly from that of CR+ and PV+. ***p < 0.001; ****p < 0.0001.

Another notable factor is the synapse number/length (μm) ratio (mean values calculated from Table 2: CB+: 0.76; CR+: 0.81; PV+: 1.18). Additionally, the mean diameter of the dendrites was determined and shown in Figure 4. Marked significant differences were observed in the mean diameter of CB + dendrites in comparison with the other two IN subtypes (CB+: 0.479 μm, CR+: 0.643 μm, and PV+: 0.699 μm; CB + vs. CR + *p* = 0.0003; CB + vs. PV + *p* < 0.0001).

Altogether, in contrast with PV, the extent of CB dendrites was the smallest, together with the amount of synaptic inputs per area. CR data were between the two markers.

### Quantitative morphometry of presynaptic boutons

**Figure 5:**
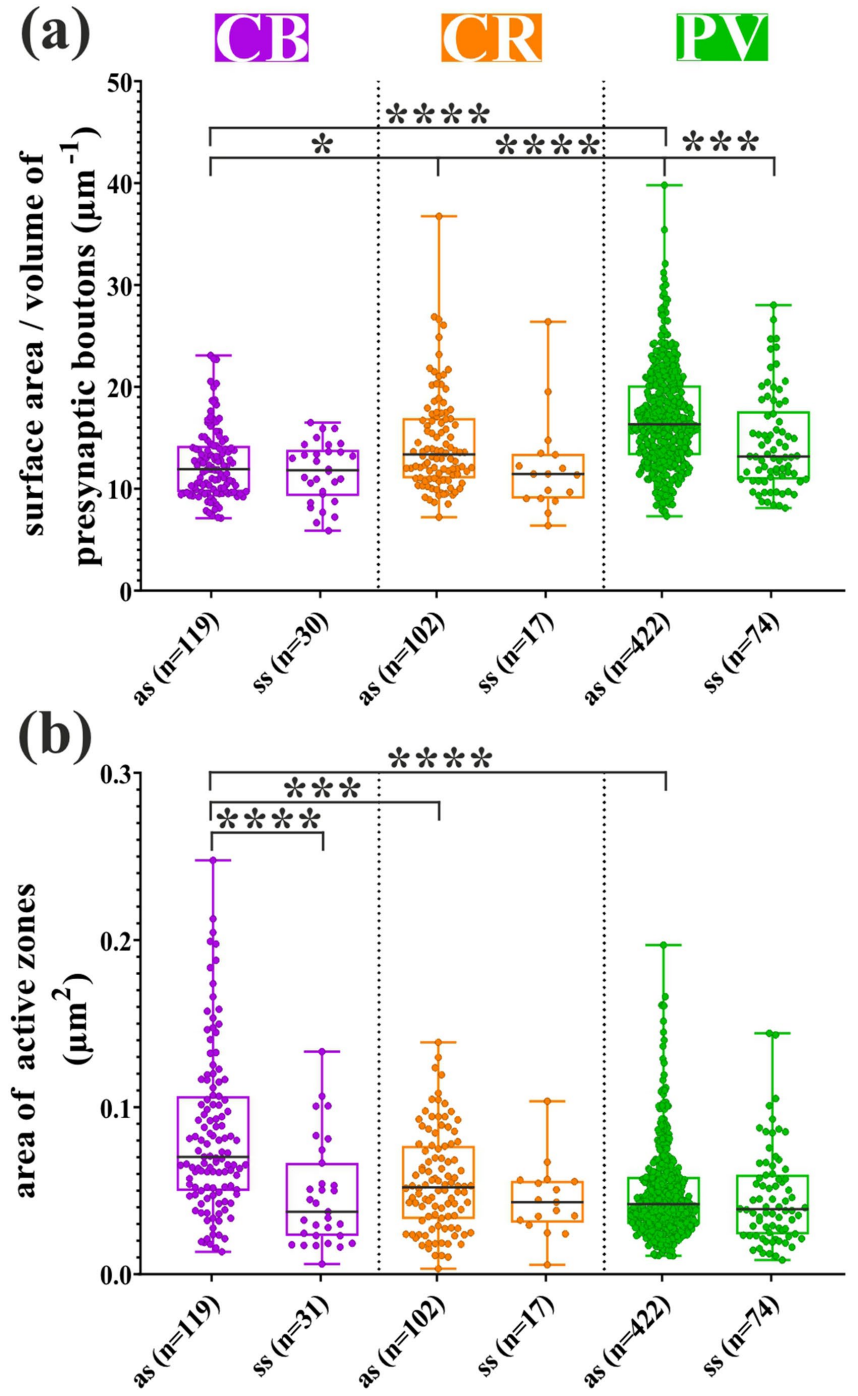
Comparisons of the main morphometric parameters of presynaptic boutons and synaptic inputs to dendrites belonging to calcium-binding protein-containing INs. **(a)** The mean size of the excitatory-type boutons (asymmetric, as) received by parvalbumin immunopositive (PV+) branches was significantly larger than those of the other two markers. In addition to this, inhibitory (symmetric, ss) boutons were significantly smaller than excitatory boutons, and this feature could not be verified statistically for the other IN subtypes. A modest, but significant difference was detected between CB+ and calretinin-immunopositive (CR+) excitatory boutons. **(b)** According to our measurements, the mean extent of the excitatory active zones was the largest in the case of CB+ dendrites and differed significantly from those of CR+ and PV+ dendrites. Symmetric synapses received by CB+ interneurons were significantly smaller than asymmetric types. However, no significant difference in the size of symmetric synapses could be detected between the three IN subtypes. *p < 0.05; ***p < 0.001; ****p < 0.0001.

To characterize the presynaptic boutons quantitatively, the ratio of bouton surface area to volume (μm^−1^) was determined and displayed in Figure 5. Boutons providing asymmetric (as) and symmetric (ss) types of synapses were distinguished for every kind of dendrite, namely, the excitatory type of boutons arriving at PV+ dendrites was significantly greater (*p* < 0.0001) than those at CB+ and CR+ (Figure 5a). In contrast, a moderate, yet significant difference was observed between CB+ and CR + INs (*p* = 0.0365). When only inhibitory boutons were considered according to the same criteria, there was no difference between the markers. Interestingly, considering cross-modal differences between the parameters of asymmetric and symmetric inputs within IN types, boutons on only PV+ dendrites revealed a significant difference (*p* = 0.0004). In contrast, in the case of the other type of dendrites, they did not.

Next, presynaptic boutons of either type (“as” and “ss”) were examined for the area extent of the active zones. Regarding the asymmetric synapses of CB+ dendrites, their active zones were significantly different from the asymmetric synapses of the other two types of dendrites (CB+ vs. CR + *p* = 0.0002; CB + vs. PV + *p* < 0.0001) (Figure 5b). In contrast, the active zone area of symmetric synapses did not differ between the three types of dendrites. Oddly enough, when symmetric and asymmetric synapses were compared within the same marker type, a significant difference was detected only for CB+ dendrites (Figure 5b, *p* < 0.0001).

### Distribution of synapses along the dendrites

**Figure 6:**
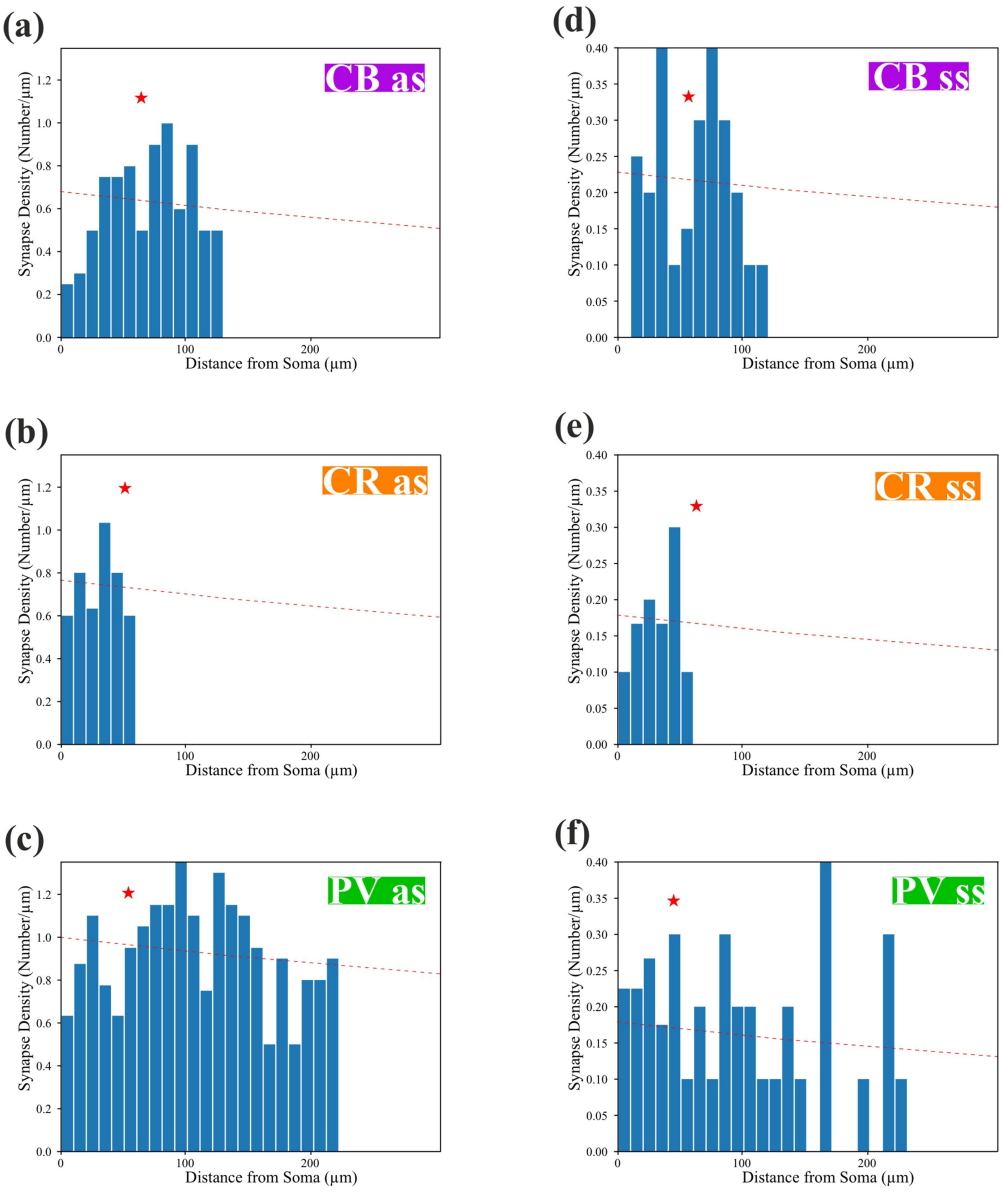
Density distribution of the synapses along the dendrites belonging to calcium-binding protein-containing interneurons. **(a–c)** The density of “as” synapses peaked proximal to the soma and decreased gradually toward the distal dendritic segments for all marker types. A fitting (red broken line) was made by using a hyperbolic function across the centroids (red star, see Materials and Methods). **(d–f)** For “ss” synapses, fitting across the centroid (red star) was less steep [as: y = 683.0816/(x + 779.8993), ss: y = 1470.8505/(x + 628.1984)] and remained clearly separated from the corresponding fit of as curve throughout the examined dendritic range, indicating a consistently lower overall density of symmetric inputs for CB-, CR-, and PV-positive cells.

Based on qualitative inspection of the reconstructed dendrites and the location of presynaptic boutons, marked differences were seen in the distribution of synapses between different IN types (Figure 3). The distance of each synapse, both excitatory and inhibitory types, was measured from the soma, and their density distribution was graphed. When data of “as” and “ss” synapses were pooled, a significant difference was detected in their distribution between the IN marker types (Figures 6a–c, CB vs. CR *p* < 0.0001; CB vs. PV *p* = 0.0004; CR vs. PV *p* < 0.0001). Considering a similar comparison for the “as” type of synapses alone, a statistically significant difference across the IN markers was found, but not in the case of symmetric synapses (CB “as” vs. CR “as” *p* < 0.0001; CB “as” vs. PV “as” *p* = 0.0017; CR “as” vs. PV “as” *p* < 0.0001). A cross-modal difference, namely, the distribution between “as” and “ss” within IN types, reached a significance level only for the PV + subtype (data not shown; “as” vs. “ss” *p* = 0.014).

For a simple comparison of the above distributions, which all showed a common tendency of decreasing the number of input synapses from the proximal toward the distal segment of the dendrites (Figures 6a–c), a hyperbolic fit (red curve) across the centroid of the distributions for each marker type was used (see Materials and Methods). The following fitting parameters for “as” and “ss” synapses were applied: “as”: *y* = 683.0082/(x + 779.8993), “ss”: *y* = 147.8505/(x + 628.1984); KS_(as)_: 0.409, p_(as)_: 0.049, KS_(ss)_: 0.421, and p_(ss)_: 0.068. In this way, an extrapolation of the density distributions toward larger distances can be made using quasi-complete IN reconstructions taken from public databases (Ascoli et al., 2007^2^^,^^3^ Allen Mouse Brain Atlas: see footnote 1).

### Clustering of the synapses

**Figure 7:**
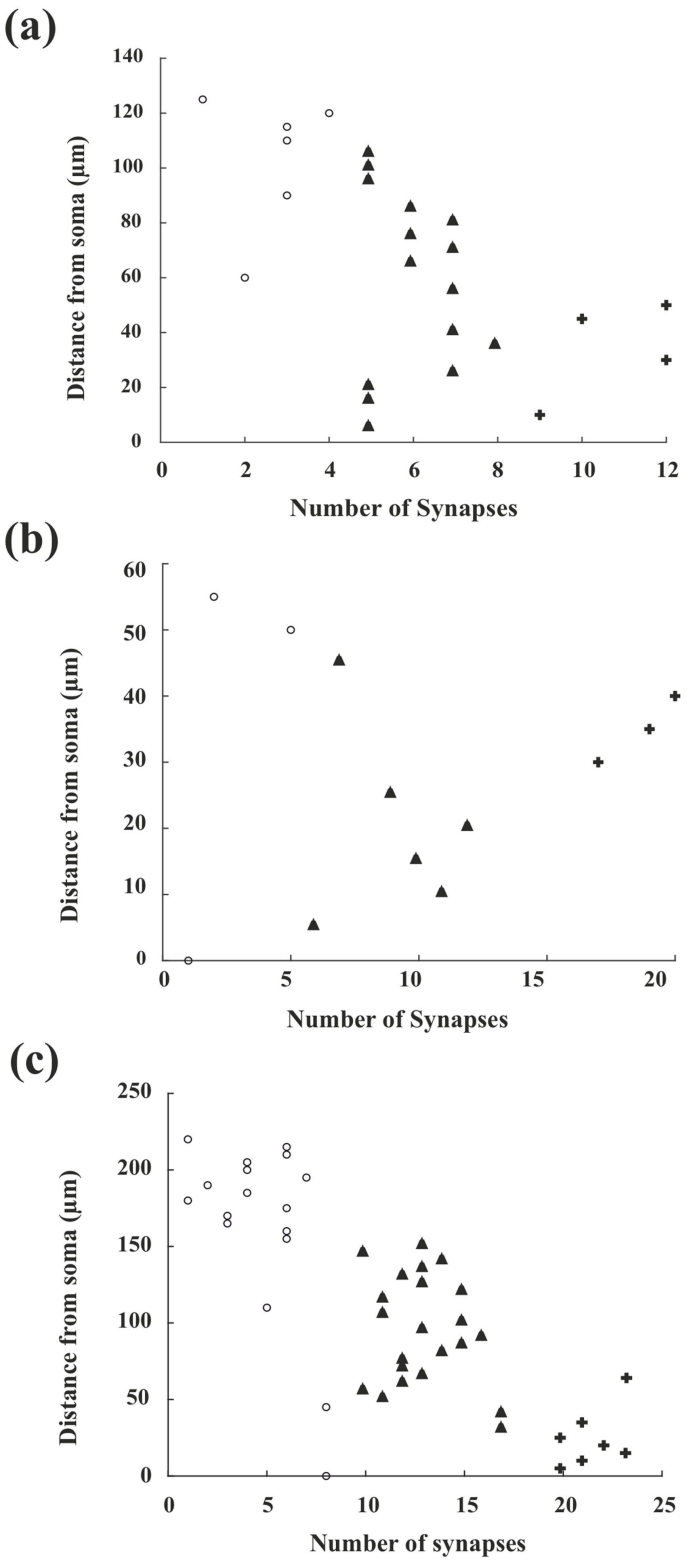
Clustering synaptic inputs along dendrites of CB+, CR+, and PV + interneurons. **(a–c)** Show scatter plots of synaptic input distribution for CB+, CR+, and PV + dendrites, respectively. The *y*-axis indicates the distance from the soma (μm), and the *x*-axis shows the number of synapses within each 5 μm bin along the dendrite. K-means clustering (with three clusters, determined via the elbow method) was applied to the binned synapse counts to detect possible spatial organization of inputs. Each symbol (open circle, triangle, cross) represents membership in one of the three clusters assigned by the K-means analysis. Thus, each point represents the number of synapses found within a specific 5-μm bin at a certain distance from the soma, as indicated by cluster affiliation. Clustering details are provided in “Materials and Methods” section. A clear separation of clusters was only apparent in the PV + group (panel c), while CB + and CR + dendrites **(a,b)** did not display distinct cluster separation.

Our primary interest was whether dendritic input is compartmentalized according to a particular attribute, whether functional or structural. In this regard, the present data offered two types of analysis. Structurally, dendritic inputs represented by putative excitatory (asymmetric synapses) and inhibitory (symmetric synapses) types were examined at a simplistic level using cluster analysis. Accordingly, the presynaptic boutons were subjected to the general K-means cluster analysis (supplemented with the elbow method), for each subtype of GABAergic INs (“as” and “ss” data were pooled), three clusters could be identified (Figures 7a–c). Afterward, the coefficient of determination (expressed by R^2^, which usually ranges from 0 to 1) was determined for the pooled dataset of each IN subtype, respectively, to test and validate our proposed model. In addition to this, Silhouette score analysis was performed to ensure validity and to provide a measure of cohesion of the predefined clusters [(−)1 indicates the worst and (+)1 indicates the best clustering quality].

First, the inputs of CB+ dendrites revealed that synaptic clusters are not separated clearly from each other. This feature is underlined by a relatively low *R*^2^ (0.3093). Silhouette scores of the data points were quite far from the best values as well (Figure 7a; Cluster 1, unfilled circles: 0.7589; Cluster 2, triangles: 0.7297; Cluster 3, crosses: 0.7983). No distinct clustering pattern was observed in the case of the CR + dendrite. Silhouette scores were highly diverse (Figure 7b; Cluster 1, 0.9452; Cluster 2, 0.5967; Cluster 3, 0.7395), and *R*^2^ was the lowest in comparison with the two other markers (0.0114). It should be noted that the sample size for the CR + group was the smallest, with only 119 synapses. The clustering pattern of input to PV + dendrites showed a clear case for which three well-defined groups of synaptic inputs could be distinguished (Figure 7c). *R*^2^ was 0.6253, and Silhouette scores were 0.8418, 0.7459, and 0.9600 for clusters 1, 2, and 3, respectively. It is worth noting that the PV+ group had the largest sample size, comprising a total of 495 synaptic input elements.

## Discussion

The present study examined the synaptic input distribution to the dendrites of CBP-containing interneurons using serial-section electron microscopy. For the nine dendrites (three dendrites of CB+, CR+, and PV+, respectively) that originated from the somata, a total of 778 presynaptic boutons were identified and reconstructed in 3D in association with the targeted dendrites. Approximately 84.1% of the boutons provided asymmetric and 15.9% symmetric synapses. Their density varied according to dendrite types, with the highest average on PV + dendrites. Regarding the distribution of all synapses (“as” and “ss”) along the dendrites, significant differences were found between the three IN types. Synapse density on PV+ dendrites was the highest with a peak closest to the soma, whereas synapses on CB+ dendrites had the lowest peak farthest to the soma (CB+: 0.6282/μm, CR+: 0.8198/μm, PV+: 1.2750/μm). Nonetheless, cross-marker differences were present for ss as well, albeit weaker than for as (e.g., CB–CR significant at α = 0.05 only), while pairs involving PV generally remained highly significant. In addition, clustering of the input synapses was present for PV+ dendrites but not for the other types.

CBP-containing interneurons comprise a substantial proportion of the GABAergic neuron population in the cerebral cortex, with an almost complete coverage (DeFelipe et al., 1999). The three main classes of CBP (CB+, PV+, and CR+) containing cells each represent a morphologically (DeFelipe, 1997; Kooijmans et al., 2020) and functionally (Zaitsev et al., 2005; Fairless et al., 2019) broad spectrum of interneurons. Despite the wealth of information that has accumulated, little is still known about the synaptic input organization onto these neurons. The lack of scarce information is particularly for the dendrites; nonetheless, there are notable attempts, for example, unraveling the density of synapses established onto dendrites of INs in the neocortex (Kawaguchi et al., 2006; Kameda et al., 2012; Hioki et al., 2013), hippocampus (Gulyás et al., 1999; Martina et al., 2000), and detailed synaptic morphometric analysis is available in mouse VISp for morphologically characterized INs (Turner et al., 2022). Here, we provide a comprehensive quantitative EM analysis of the synaptic coverage of neurochemically identified CBP-containing INs supplemented with a conservative estimate of their total synaptic supply based on IN reconstructions from available databases (Ascoli et al., 2007, see footnote 2, 3).

### Sample selection for EM analysis

A general feature of cortical inhibitory neurons is that their dendrites are thin, typically not exceeding 1 μm in diameter (see Figure 5), and are even close to the parent soma (Emri et al., 2001). This is in stark contrast with the dendrites of pyramidal cells, whose apical dendrite is several μm thick, even hundreds of μm away from the cell body. Following this, interneuron dendrites pose a challenge for EM investigations as well as neurophysiological recordings, due to their small size.

Although CB+ -containing interneurons are present in all layers of the visual cortex, they are persistent in L2/3 and L5 (Park et al., 2002). CR + INs form the most frequent subtype in L2/3 with 42% (Gonchar et al., 2008). The PV + INs constitute the densest subtype in L5, but although they are present in L2/3, which includes basket cells and chandelier cells (Gonchar et al., 2008; Lee et al., 2010; Xu et al., 2010). Based on the abovementioned laminar distribution of the different CBP subtypes for the EM analysis, we chose the particular neuron from the cortical layer in which the corresponding subtype has the highest representation. It should be emphasized that our EM analysis had to be restricted to a limited number of dendrites due, primarily, to technical reasons (section thickness).

### Synaptic coverage of interneuron dendrites

It is well known that the intrinsic conductance of dendrites is proportional to both the dendritic surface area and the extension of the synaptic surfaces, and these factors are crucial for computational modeling of the dendrites (Turner et al., 2022). Accordingly, numerous morphological parameters such as dendritic diameter and branching pattern influence signal generation and propagation (van Elburg and van Ooyen, 2010; de Sousa et al., 2015). Furthermore, intracellular calcium concentration [Ca^2+^]i can influence the plasticity of synapses and, in turn, can regulate activity-dependent plasticity mechanisms of the neocortex (long-term potentiation, LTP, and long-term depression, LTD) (Bar-Ilan et al., 2013). Taking into account the factors mentioned above, we measured the mean diameter (Figure 4) of the main dendritic shafts of the designated CBP-containing INs as an important indicator also for dendritic volume. Our data corresponded well with those of recent studies obtained in mice (Kooijmans et al., 2020; Medalla et al., 2023).

### The ratio of excitatory versus inhibitory synapses on IN dendrites

Morphometric and physiological parameters of synaptic inputs are essential for mapping neuronal connectivity (Hildebrand et al., 2017; Iacaruso et al., 2017; Meijering et al., 2016; Sigal et al., 2015). For example, presynaptic boutons were determined using volume-based electron microscopic sorting in the mouse thalamus (Maher et al., 2023). Another study distinguished subtle differences in the ultrastructural features of presynaptic boutons of presumed excitatory and inhibitory types impinging on the dendrites of an inhibitory cell type (Ahmed et al., 1997) to map out synapses along the dendrites. These studies exploited structural features to infer functional considerations. The plasticity status of each excitatory synapse, namely, “protected” or unchanged, can be potentiated or depressed, depending on the distance from the neighboring inhibitory inputs (Bar-Ilan et al., 2013). Additionally, alterations in plasticity within the dendritic microdomains can lead to disruptions in the dendritic integration of individual neurons (Ravasenga et al., 2022). Nevertheless, basic structural parameters of individual GABAergic INs as well as different IN subtypes are still not fully available: (i) E/I balance of dendritic segments and microdomains, (ii) exact location of synaptic active zones, and (iii) surface extension of synaptic active zones at the nanometer level. Our previously elaborated LM-correlated EM method enabled us to collect these datasets, for the first time, for CBP-containing INs in the mouse VISp. Among the three subtypes, PV + INs were salient with the quantitative morphometry of excitatory presynaptic boutons, and significant differences between excitatory and inhibitory types were detected only in this subgroup, too. Excitatory presynaptic boutons that ended onto CB+ and CR+ dendrites were significantly smaller than those that ended on PV+ ones. Nevertheless, these features were characteristic only of excitatory inputs; the surface extent/volume ratio of inhibitory synapses was quite the same between the neurochemical markers (Figure 5a). Interestingly, the surface area of excitatory active zones showed the opposite pattern, with the inputs of the CB+ dendrites being measured to be the most extensive, while those converging onto PV+ dendrites were the smallest (Figure 5b). However, differences in inhibitory synapses were not found when the three markers were compared to each other.

Because this study determined the number and location of excitatory and inhibitory synapses along the dendrites, the ratio of E/I synapses for the three CBP types could also be calculated (Table 2). The widely used phenomenon, E/I balance, can refer to very different aspects of nerve cell function depending on the perspective of the investigation, but in general describes the ability of the neuron to keep its overall firing pattern in a narrow range (Eichler and Meier, 2008) applying different regulatory mechanisms of excitatory and inhibitory inputs in the PCs and INs (Spiegel et al., 2014). The term E/I balance can demonstrate the subset of the inhibitory inputs derived from a specific IN subtype, but mostly it means the total number of inhibitory synapses received by an individual neuron (He and Cline, 2019). Here, we use a simplified version of the latter definition, namely, quantifying the global structural information about excitatory and inhibitory inputs to the IN subtypes. Our quantitative dataset supports this notion as significant differences were found between the morphometry (see Figure 5) and distribution of synapses (see Figures 6, 7) comparing the three CBP-containing IN subtypes.

### Total number of synaptic inputs to CBP-containing interneurons

Dendritic input: Inhibitory neurons are essential components of the cortical circuit whose function is determined by synaptic inputs chiefly arriving on the dendrites. In this regard, a fundamental question that arises is “what the total amount of synapses to a single IN is and what proportion of “as” and “ss” types they represent.” We attempted to answer the above questions for the CBP subtypes, even though our dendritic EM survey is inherently constrained by a several limiting factors, such as technical limitations and limited resources. To estimate the total number of synapses, we used fitting functions to our EM data and applied them to single-neuron reconstructions of CB+, CR+, and PV+ IN types available in public databases (see Supplementary Table 1). First, we selected intracellularly stained neurons from those cortical layers in which they are typically found. Second, a dendrogram was generated of each neuron reconstruction (Supplementary Figure 1), which allowed for determining the total dendritic length and branching positions with respect to soma origin. Third, we utilized the synapse density function (hyperbolic, Figure 6) obtained for the EM reconstruction of designated CBP interneuron types. According to the available data bases the average dendritic length was 2,593 μm (CB+), 2,584 μm (CR+), and 2,352 μm (PV+) and which according to our synapse density measurements corresponds to a total of 2,102 (1,572 “as”; 531 “ss”), 2,044 (1,675 “as”; 368 “ss”), and 2,674 (2,305 “as”; 503 “ss”) synapses, respectively (Supplementary Table 1). At this point, several aspects need to be discussed when comparing the above values with those of previous studies. Notably, the total number of dendritic synapses calculated here for neocortical INs differed by a factor of 0.609 (CB+, 3,585), 1.159 (CR+, 2,043), and 0.199 (PV+, 15,692) compared with the inhibitory neuron types of the hippocampus (see Table 5 in Gulyás et al., 1999) even though the dendritic length values in the latter study [3,441 μm (CB+), 2,499 μm (CR+), 4,347 μm (PV+), see Table 2 in Gulyás et al., 1999] were somewhat similar to those reported here (Supplementary Table 1). The only notable difference was in the category of hippocampal PV+ dendrites, which received almost 5 times more synapses than their neocortical counterparts. Furthermore, the amount of dendritic input across the neocortical CBP types differed less than that of the archicortical counterparts (see Supplementary Table 1). This may reflect a genuine organizational difference between the two brain regions, particularly, for PV+ cells, many of which represent basket cells.

Total input: To get a complete view of the total number of synaptic inputs to a neuron type one should take into account synapses terminating on the cell body in addition to the dendrites. While the present study focused on the number and layout of synapses in the dendritic processes, the somatic input to CBP-containing INs was estimated based on EM data obtained in the hippocampus (Gulyás et al., 1999). They found that somatic synapses accounted for only a small proportion of all synapses (CB+, 6.36%; CR+, 6.13%; PV+, 3.00%). Applying the above data and extending onto neocortical CBP-containing INs, a conservative estimate for the total number of synapses could be made: 2325 (CB+); 2,526 (CR+); 3,224 (PV+). Taken together, according to the above calculation, neocortical CBP INs receive somewhat fewer synapses than their archicortical counterparts.

### Arrangement and clustering of dendritic synapses

It is well known that each IN subtype is involved in different aspects of the responses established in cortical circuits. Notably, different INs with unique response characteristics can result in feature-selective responses (Mao et al., 2012). For example, long-range projections from different cortical areas recruit specific subtypes of GABAergic INs in primary sensory cortices (Naskar et al., 2021). In this regard, the arrangement of synaptic inputs originating from different sources along the dendrite is pivotal in determining neuronal response characteristics (Fişek et al., 2023). Analyses of the synaptic composition of dendrites are typically restricted only to a few μm length from which most numerical values are inferred (Merchán-Pérez et al., 2009; Montero-Crespo et al., 2020; Ahmed et al., 1994; Gulyás et al., 1999). Direct mapping of the complete synaptic input of dendrites (dendrome) at the ultrastructural level is, consequently, of prime interest. In this regard, theoretical studies are available for spiny excitatory cells using conductance-based compartmental neuronal models (Segev et al., 1995; Eyal et al., 2014, 2018). These studies indicate that individual neurons are likely to receive semi-random dendritic input, rather than clustered input on specific dendrites (Amsalem et al., 2020). For inhibitory neurons, available functional data indicate that differential input constellation on the dendrites can explain cell-type-specific behavior and activity patterns (Kohus et al., 2016). According to our results, the presynaptic inputs to the dendrites of CBP-containing IN subtypes are organized into three clusters; however, only in the case of PV+ cells, a clear correlation detected with distance from the soma. It is tempting to ask whether the clustering of synaptic input does reflect a functional role, e.g., triggering local nonlinearities (Ujfalussy and Makara, 2020). Interestingly, functional imaging studies have yielded controversial results in terms of dendritic input compartmentalization (Chen et al., 2011; but see Kerlin et al., 2019; Ujfalussy and Makara, 2020). The clustering of presynaptic bouton types that we observed, in particular for PV + INs, may well represent the structural underpinnings of such functions. Clearly, further studies are needed to elucidate the significance of dendritic input clustering.

## Conclusion

Setting out computational models of cortical IN types is an effective tool for exploring their functional role. One of the key points is the complete characterization of the presynaptic inputs to dendrites. Although we are still far from this, this study provides an essential dataset regarding the synaptome of the CBP-containing INs in mouse VISp. Morphometric features of presynaptic inputs with excitatory characteristics, but not inhibitory ones, terminating on CB+, CR+, and PV+ dendrites were significantly different. The clustering of synapses along the dendrites could be seen only for PV+ cells. These results show a complex organization of synaptic input to IN dendrites with common tendencies across subtypes. The data presented here provide structural information that can be exploited in computational models to enhance the understanding of synaptic integration in IN dendrites.

## Data Availability

The original contributions presented in the study are included in the article/Supplementary material, further inquiries can be directed to the corresponding author/s.

## Footnotes

^1^ https://celltypes.brain-map.org/overview

^2^ https://NeuroMorpho.org

^3^ http://celltypes.brain-map.org/

## Supplementary material

SUPPLEMENTARY TABLE 1 Synaptic density and total synaptic coverage of dendrites of intracellularly labelled cells in visual cortices of mice. For the calculations, neuron reconstructions were downloaded from publicly available databases (Ascoli et al, 2007, https://NeuroMorpho.org; http://celltypes.brain-map.org). as: asymmetric synapse, ss: symmetric synapse.

SUPPLEMENTARY FIGURE 1 Assessment of the dendritic synaptome of CB+, CR+, and PV+ subtypes of INs. For each subtype, a single example is shown of the neuron reconstruction pool (see Supplementary Table 1). The left column displays a light microscopic reconstruction of dendrites of intracellularly labelled IN subtypes. The URL link (in white) helps to correlate each cell with the database origin. The second column displays dendrograms of the reconstructed INs, indicating the distances from the soma origin to the terminal end node (N) of each dendrite. The third column represents the frequency distribution of dendritic segments as a function of distance from their soma origin. The fourth column shows the distribution of excitatory (as) and inhibitory (ss) synapses for the entire dendritic tree of the selected INs. Stars indicate maxima of as (blue) and ss (orange) synapses. The number of synapses (as and ss) was determined on the basis of the EM reconstructions (see Figures 3, 6) and extrapolated for the intracellularly reconstructed INs obtained from the databases.
