# Supplementary material for: Dendritic synaptome of calcium-binding protein containing GABAergic interneurons in the mouse primary visual cortex
Source: Front Neural Circuits. 2025 Oct 8;19:1644572. doi: 10.3389/fncir.2025.1644572 (PMC12540436; doi:10.3389/fncir.2025.1644572)
Supplement: SUPPLEMENTARY TABLE 1 — Synaptic density and total synaptic coverage of dendrites of intracellularly labelled cells in visual cortices of mice. For the calculations, neuron reconstructions were downloaded from publicly available databases (Ascoli et al, 2007, https://NeuroMorpho.org; http://celltypes.brain-map.org). as: asymmetric synapse, ss: symmetric synapse. [file Table_1.docx]

|  |  |  |  |  |  |  |  |  |  |  |
| --- | --- | --- | --- | --- | --- | --- | --- | --- | --- | --- |
| **Calb+ Cell ID** | **Mouse Line** | **Area** | **Layer** | **Link** | **Name of file** | **Number of asymmetric synapses** | **Number of symmetric synapses** | **Number of all synapses** | **Length of dendrites (um)** | **Density (synapses/length)** |
| NMO_276055 | Calb2-IRES-Cre | V1 | 1 | https://neuromorpho.org/neuron_info.jsp?neuron_name=842841654_transformed | 842841654_transformed.CNG.swc | 1910 | 643 | 2553 | 3066 | 0,833 |
| **Average** |  |  | **1** |  |  | **1910** | **643** | **2553** | **3066** | **0,833** |
| **Std** |  |  | **1** |  |  | **N/A** | **N/A** | **N/A** | **N/A** |  |
|  |  |  |  |  |  |  |  |  |  |  |
| NMO_276273 | Calb2-IRES-Cre | LM | 2/3 | <https://neuromorpho.org/neuron_info.jsp?neuron_name=841834394_transformed> | 841834394_transformed.CNG.swc | 2170 | 733 | 2903 | 3631 | 0,799 |
| **Average** |  |  | **2/3** |  |  | **2170** | **733** | **2903** | **3631** | **0,799** |
| **Std** |  |  | **2/3** |  |  | **N/A** | **N/A** | **N/A** | **N/A** |  |
|  |  |  |  |  |  |  |  |  |  |  |
| NMO_276889 | Calb2-IRES-Cre | V1 | 4 | https://neuromorpho.org/neuron_info.jsp?neuron_name=745736817_transformed | 745736817_transformed.CNG.swc | 1585 | 534 | 2119 | 2555 | 0,829 |
| NMO_276981 | Calb2-IRES-Cre | PM | 4 | https://neuromorpho.org/neuron_info.jsp?neuron_name=841718359_transformed | 841718359_transformed.CNG.swc | 1170 | 395 | 1565 | 1913 | 0,818 |
| NMO_276006 | Calb2-IRES-Cre | V1 | 4 | https://neuromorpho.org/neuron_info.jsp?neuron_name=842844583_transformed | 842844583_transformed.CNG.swc | 1280 | 432 | 1711 | 2099 | 0,815 |
| **Average** |  |  | **4** |  |  | **1345** | **454** | **1798** | **2189** | **0,822** |
| **Std** |  |  | **4** |  |  | **215** | **72** | **287** | **330** |  |
|  |  |  |  |  |  |  |  |  |  |  |
| NMO_276985 | Calb2-IRES-Cre | PM | 5 | https://neuromorpho.org/neuron_info.jsp?neuron_name=746257405_transformed | 746257405_transformed.CNG.swc | 1740 | 588 | 2328 | 2913 | 0,799 |
| NMO_276001 | Calb2-IRES-Cre | V1 | 5 | https://neuromorpho.org/neuron_info.jsp?neuron_name=746754997_transformed | 746754997_transformed.CNG.swc | 1200 | 405 | 1605 | 1960 | 0,819 |
| NMO_275995 | Calb2-IRES-Cre | LM | 5 | https://neuromorpho.org/neuron_info.jsp?neuron_name=847083836_transformed | 847083836_transformed.CNG.swc | 1519 | 515 | 2033 | 2609 | 0,779 |
| **Average** |  |  | **5** |  |  | **1486** | **503** | **1989** | **2494** | **0,797** |
| **Std** |  |  | **5** |  |  | **272** | **92** | **364** | **487** |  |
| **Grand Average** |  |  | **Pooled** |  |  | **1572** | **531** | **2102** | **2593** | **0.811** |
| **Std** |  |  | **Pooled** |  |  | **363** | **124** | **487** | **599** |  |
|  |  |  |  |  |  |  |  |  |  |  |
| **VIP+ Cell ID** | **Mouse Line** | **Area** | **Layer** | **Link** | **Name of file** | **Number of asymmetric synapses** | **Number of symmetric synapses** | **Number of all synapses** | **Length of dendrites (um)** | **Density (synapses/length)** |
| 580145037 | Vip-IRES-Cre | PM | 2/3 | http://celltypes.brain-map.org/experiment/morphology/580145037 | Vipr2-IRES2-Cre_Ai14-310513.05.02.01_637021223_m.swc | 1513 | 342 | 1855 | 2170 | 0,855 |
| 569072334 | Vip-IRES-Cre | AL | 2/3 | http://celltypes.brain-map.org/experiment/morphology/569072334 | Vipr2-IRES2-Cre_Ai14-297026.05.02.01_759940062_m.swc | 1472 | 333 | 1805 | 2103 | 0,858 |
| 569810649 | Vip-IRES-Cre | V1 | 2/3 | http://celltypes.brain-map.org/experiment/morphology/569810649 | Vip-IRES-Cre_Ai14-299124.04.02.01_759934589_m.swc | 1645 | 363 | 2007 | 2523 | 0,796 |
| 570896413 | Vip-IRES-Cre | V1 | 2/3 | http://celltypes.brain-map.org/experiment/morphology/570896413 | Vip-IRES-Cre_Ai14-299123.04.01.01_587405140_m.swc | 1294 | 290 | 1584 | 1899 | 0,834 |
| 567007144 | Vip-IRES-Cre | V1 | 2/3 | http://celltypes.brain-map.org/experiment/morphology/567007144 | Vip-IRES-Cre_Ai14-294014.04.02.01_659296496_m.swc | 1215 | 266 | 1482 | 1901 | 0,779 |
| 535708196 | Vip-IRES-Cre | V1 | 2/3 | http://celltypes.brain-map.org/experiment/morphology/535708196 | Vip-IRES-Cre_Ai14_IVSCC_-264875.04.02.01_559392178_m.swc | 2154 | 475 | 2629 | 3297 | 0,797 |
| 524689239 | Vip-IRES-Cre | V1 | 2/3 | http://celltypes.brain-map.org/experiment/morphology/524689239 | Vip-IRES-Cre_Ai14_IVSCC_-253382.05.01.01_556384349_m.swc | 2307 | 505 | 2812 | 3590 | 0,783 |
| 514767977 | Vip-IRES-Cre | V1 | 2/3 | http://celltypes.brain-map.org/experiment/morphology/514767977 | Vip-IRES-Cre_Ai14_IVSCC_-247244.05.02.01_657210312_m.swc | 1461 | 322 | 1783 | 2234 | 0,798 |
| **Average** |  |  | **2/3** |  |  | **1633** | **362** | **1995** | **2465** | **0,809** |
| **Std** |  |  | **2/3** |  |  | **394** | **85** | **478** | **640** |  |
|  |  |  |  |  |  |  |  |  |  |  |
| 569809287 | Vip-IRES-Cre | V1 | 4 | http://celltypes.brain-map.org/experiment/morphology/569809287 | Vip-IRES-Cre_Ai14-299124.05.01.01_591276089_m.swc | 2048 | 444 | 2492 | 3288 | 0,758 |
| 561934585 | Vip-IRES-Cre | V1 | 4 | http://celltypes.brain-map.org/experiment/morphology/561934585 | Vip-IRES-Cre_Ai14-287858.04.01.01_596793095_m.swc | 2689 | 584 | 3272 | 4270 | 0,766 |
| 535728342 | Vip-IRES-Cre | V1 | 4 | http://celltypes.brain-map.org/experiment/morphology/535728342 | Vip-IRES-Cre_Ai14_IVSCC_-264875.06.01.01_653337413_m.swc | 1080 | 231 | 1312 | 1784 | 0,735 |
| 515315072 | Vip-IRES-Cre | V1 | 4 | http://celltypes.brain-map.org/experiment/morphology/515315072 | Vip-IRES-Cre_Ai14_IVSCC_-243617.05.02.01_657267317_m.swc | 1520 | 332 | 1852 | 2384 | 0,777 |
| **Average** |  |  | **4** |  |  | **1834** | **398** | **2232** | **2932** | **0,761** |
| **Std** |  |  | **4** |  |  | **694** | **151** | **845** | **1086** |  |
|  |  |  |  |  |  |  |  |  |  |  |
| 590558808 | Vip-IRES-Cre | PM | 6 | http://celltypes.brain-map.org/experiment/morphology/590558808 | Vipr2-IRES2-Cre_Ai14-316908.04.02.01_648577094_m.swc | 1448 | 325 | 1773 | 2111 | 0,840 |
| 579662957 | Vip-IRES-Cre | V1 | 6 | http://celltypes.brain-map.org/experiment/morphology/579662957 | Vipr2-IRES2-Cre_Ai14-310515.06.01.01_596799483_m.swc | 577 | 132 | 709 | 792 | 0,895 |
| 573404307 | Vip-IRES-Cre | V1 | 6 | http://celltypes.brain-map.org/experiment/morphology/573404307 | Vipr2-IRES2-Cre_Ai14-304184.04.01.01_601947998_m.swc | 2416 | 523 | 2938 | 3879 | 0,757 |
| 582917630 | Vip-IRES-Cre | V1 | 6 | http://celltypes.brain-map.org/experiment/morphology/582917630 | Vip-IRES-Cre_Ai14-313529.03.01.01_596799462_m.swc | 2631 | 574 | 3205 | 4132 | 0,776 |
| 570896453 | Vip-IRES-Cre | V1 | 6 | http://celltypes.brain-map.org/experiment/morphology/570896453 | Vip-IRES-Cre_Ai14-299123.03.01.01_599475378_m.swc | 1429 | 311 | 1740 | 2252 | 0,773 |
| 565880475 | Vip-IRES-Cre | V1 | 6 | http://celltypes.brain-map.org/experiment/morphology/565880475 | Vip-IRES-Cre_Ai14-294013.04.02.01_591275914_m.swc | 1259 | 280 | 1539 | 1895 | 0,812 |
| **Average** |  |  | **6** |  |  | **1626** | **357** | **1984** | **2510** | **0,790** |
| **Std** |  |  | **6** |  |  | **767** | **164** | **930** | **1270** |  |
| **Grand Average** |  |  | **Pooled** |  |  | **1675** | **368** | **2044** | **2584** | **0.791** |
| **Std** |  |  | **Pooled** |  |  | **613** | **137** | **750** | **942** |  |
|  |  |  |  |  |  |  |  |  |  |  |
| **PV+ Cell ID** | **Mouse Line** | **Area** | **Layer** | **Link** | **Name of file** | **Number of as synapses** | **Number of ss synapses** | **Number of all synapses** | **Length of dendrites (um)** | **Density of synapses (1/um)** |
| 574992320 | Pvalb-IRES-Cre | V1 | 2/3 | https://celltypes.brain-map.org/experiment/morphology/574992320 | Pvalb-IRES-Cre_Ai14-305768.04.02.01_656999337_m.swc | 2626 | 460 | 3086 | 2651 | 1,164 |
| 571379222 | Pvalb-IRES-Cre | V1 | 2/3 | https://celltypes.brain-map.org/experiment/morphology/571379222 | Pvalb-IRES-Cre_Ai14-299810.04.02.01_638824825_m.swc | 2640 | 457 | 3097 | 2703 | 1,146 |
| 567952169 | Pvalb-IRES-Cre | V1 | 2/3 | https://celltypes.brain-map.org/experiment/morphology/567952169 | Pvalb-IRES-Cre_Ai14-296193.04.01.01_596792650_m.swc | 3393 | 586 | 3979 | 3488 | 1,141 |
| 567927838 | Pvalb-IRES-Cre | V1 | 2/3 | https://celltypes.brain-map.org/experiment/morphology/567927838 | Pvalb-IRES-Cre_Ai14-296193.03.01.02_650175812_m.swc | 1692 | 295 | 1986 | 1726 | 1,151 |
| 509515969 | Pvalb-IRES-Cre | V1 | 2/3 | https://celltypes.brain-map.org/experiment/morphology/509515969 | Pvalb-IRES-Cre_Ai14-236447.02.01.01_657270169_m.swc | 1793 | 316 | 2109 | 1795 | 1,175 |
| 488501071 | Pvalb-IRES-Cre | V1 | 2/3 | https://celltypes.brain-map.org/experiment/morphology/488501071 | Pvalb-IRES-Cre_Ai14-212440.05.02.01_657246943_m.swc | 2009 | 351 | 2359 | 2037 | 1,158 |
| 485184849 | Pvalb-IRES-Cre | V1 | 2/3 | https://celltypes.brain-map.org/experiment/morphology/485184849 | Pvalb-IRES-Cre_Ai14-202470.03.02.01_657298379_m.swc | 2413 | 426 | 2838 | 2410 | 1,178 |
| 469992918 | Pvalb-IRES-Cre | V1 | 2/3 | https://celltypes.brain-map.org/experiment/morphology/469992918 | Pvalb-IRES-Cre_Ai14-180631.02.01.01_501276725_m.swc | 2853 | 499 | 3352 | 2885 | 1,162 |
| 341442651 | Pvalb-IRES-Cre | V1 | 2/3 | https://celltypes.brain-map.org/experiment/morphology/341442651 | Pvalb-IRES-Cre_Ai14-178310.02.01.01_657253247_m.swc | 2669 | 474 | 3143 | 2651 | 1,186 |
| **Average** |  |  | **2/3** |  |  | **2454** | **429** | **2883** | **2483** | **1,161** |
| **Std** |  |  | **2/3** |  |  | **544** | **94** | **638** | **562** |  |
|  |  |  |  |  |  |  |  |  |  |  |
| 591268268 | Pvalb-IRES-Cre | V1 | 5 | https://celltypes.brain-map.org/experiment/morphology/591268268 | Pvalb-IRES-Cre_Ai14-321032.03.02.01_657879305_m.swc | 1606 | 276 | 1882 | 1660 | 1,134 |
| 574377552 | Pvalb-IRES-Cre | V1 | 5 | https://celltypes.brain-map.org/experiment/morphology/574377552 | Pvalb-IRES-Cre_Ai14-304103.03.02.01_623892110_m.swc | 2420 | 420 | 2839 | 2473 | 1,148 |
| 572609108 | Pvalb-IRES-Cre | V1 | 5 | https://celltypes.brain-map.org/experiment/morphology/572609108 | Pvalb-IRES-Cre_Ai14-301945.04.01.01_657409634_m.swc | 1759 | 303 | 2062 | 1816 | 1,135 |
| 569998790 | Pvalb-IRES-Cre | V1 | 5 | https://celltypes.brain-map.org/experiment/morphology/569998790 | Pvalb-IRES-Cre_Ai14-299333.03.01.01_596792857_m.swc | 3010 | 520 | 3529 | 3088 | 1,143 |
| 490387590 | Pvalb-IRES-Cre | V1 | 5 | https://celltypes.brain-map.org/experiment/morphology/490387590 | Pvalb-IRES-Cre_Ai14-215471.02.01.01_514342400_m.swc | 2008 | 2097 | 4105 | 2146 | 1,913 |
| 481093525 | Pvalb-IRES-Cre | V1 | 5 | https://celltypes.brain-map.org/experiment/morphology/481093525 | Pvalb-IRES-Cre_Ai14-198335.04.01.01_653182186_m.swc | 2461 | 425 | 2886 | 2530 | 1,141 |
| 396608557 | Pvalb-IRES-Cre | V1 | 5 | https://celltypes.brain-map.org/experiment/morphology/396608557 | Pvalb-IRES-Cre_Ai14-177452.03.02.01_673116803_m.swc | 1542 | 272 | 1814 | 1542 | 1,176 |
| 333785962 | Pvalb-IRES-Cre | V1 | 5 | https://celltypes.brain-map.org/experiment/morphology/333785962 | Pvalb-IRES-Cre_Ai14-176852.05.02.01_513557613_m.swc | 2299 | 377 | 394 | 2392 | 0,165 |
| **Average** |  |  | **5** |  |  | **2138** | **586** | **2439** | **2206** | **1,106** |
| **Std** |  |  | **5** |  |  | **709** | **600** | **1237** | **732** |  |
| **Grand Average** |  |  | **Pooled** |  |  | **2305** | **503** | **2674** | **2352** | **1.368** |
| **Std** |  |  | **Pooled** |  |  | **509** | **87** | **596** | **544** |  |
|  |  |  |  |  |  |  |  |  |  |  |
