# Supplementary material for: Dendritic synaptome of calcium-binding protein containing GABAergic interneurons in the mouse primary visual cortex
Source: Front Neural Circuits. 2025 Oct 8;19:1644572. doi: 10.3389/fncir.2025.1644572 (PMC12540436; doi:10.3389/fncir.2025.1644572)
Supplement: SUPPLEMENTARY FIGURE 1 — Assessment of the dendritic synaptome of CB+, CR+, and PV+ subtypes of INs. For each subtype, a single example is shown of the neuron reconstruction pool (see Supplementary Table 1). The left column displays a light microscopic reconstruction of dendrites of intracellularly labelled IN subtypes. The URL link (in white) helps to correlate each cell with the database origin. The second column displays dendrograms of the reconstructed INs, indicating the distances from the soma origin to the terminal end node (N) of each dendrite. The third column represents the frequency distribution of dendritic segments as a function of distance from their soma origin. The fourth column shows the distribution of excitatory (as) and inhibitory (ss) synapses for the entire dendritic tree of the selected INs. Stars indicate maxima of as (blue) and ss (orange) synapses. The number of synapses (as and ss) was determined on the basis of the EM reconstructions (see Figures 3, 6) and extrapolated for the intracellularly reconstructed INs obtained from the databases. [file Supplementary_file_1.pdf]

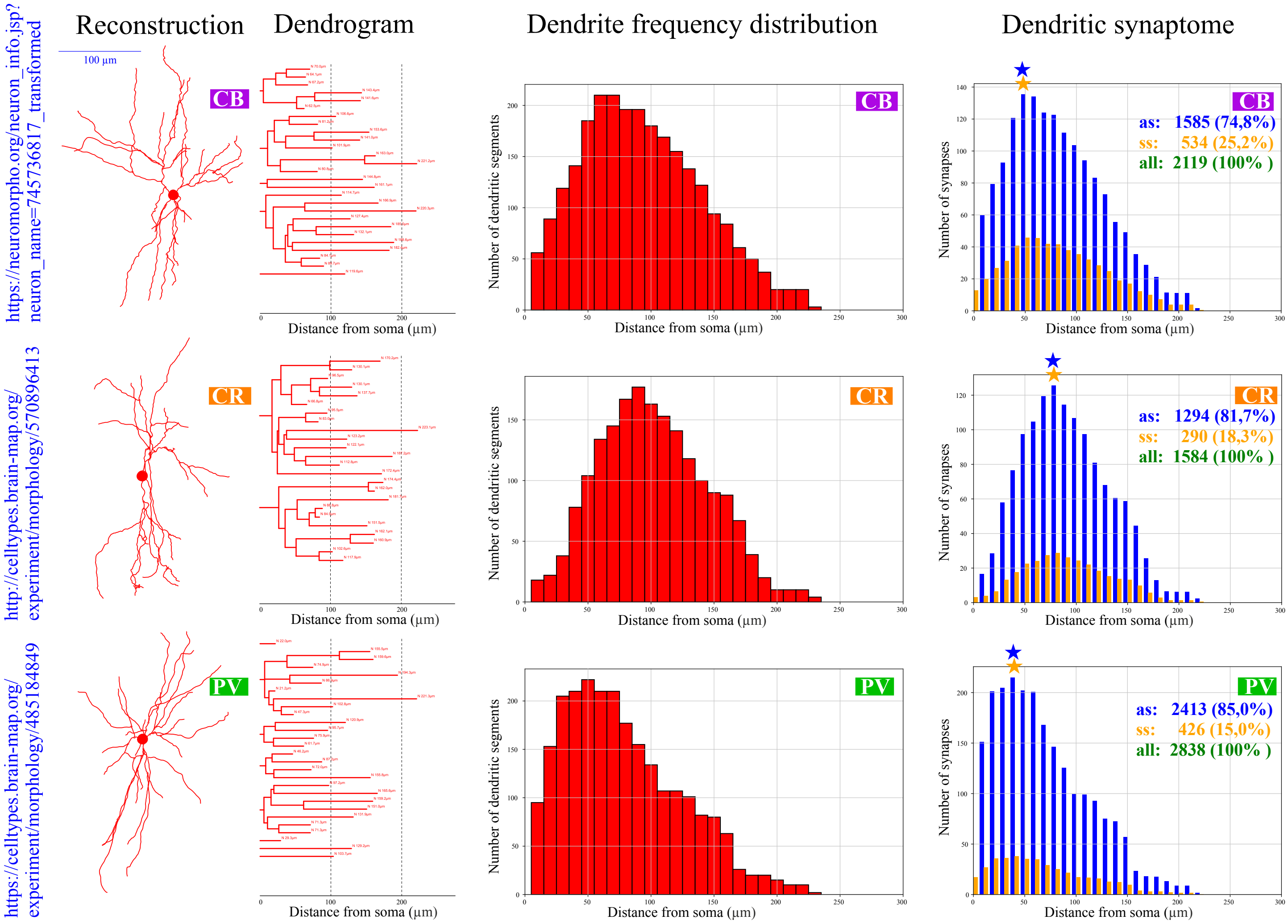
Reconstruction
Dendrogram
Dendrite frequency distribution
Dendritic synaptome
100 µm
https://neuromorpho.org/neuron_info.jsp?neuron_name=74573681_transformed
http://celltypes.brain-map.org/experiment/morphology/570896413
https://celltypes.brain-map.org/experiment/morphology/485184849
CB
CR
PV
Distance from soma (µm)
Number of dendritic segments
Number of synapses
as: 1585 (74,8%)
ss: 534 (25,2%)
all: 2119 (100%)
as: 1294 (81,7%)
ss: 290 (18,3%)
all: 1584 (100%)
as: 2413 (85,0%)
ss: 426 (15,0%)
all: 2838 (100%)
